# Resonant-Type Piezoelectric Pump Driven by Piezoelectric Stacks and a Rhombic Micro Displacement Amplifier

**DOI:** 10.3390/mi14091764

**Published:** 2023-09-13

**Authors:** Chunli Zhu, Xiaolong Shu, Dongcai Liu, Xianghan Du, Lexi Li, Qiaosheng Pan

**Affiliations:** 1 Anhui Province Key Laboratory of Simulation and Design for Electronic Information System, Hefei Normal University, Hefei 230601, China; 2 Anhui Province Key Laboratory of Measuring Theory and Precision Instrument, School of Instrument Science and Optoelectronics Engineering, Hefei University of Technology, Hefei 230009, China

**Keywords:** piezoelectric pump, resonant-type, displacement amplifier, piezoelectric stack

## Abstract

To obtain a high flow rate, a resonant-type piezoelectric pump is designed, fabricated, and studied in this paper. The pump consists of four parts: a piezoelectric vibrator, a pump chamber, a check valve and a compressible space. The designed piezoelectric vibrator is composed of a rhombic micro displacement amplifier, counterweight blocks and two piezoelectric stacks with low-voltage drive and a large output displacement. ANSYS software (Workbench 19.0) simulation results show that at the natural frequency of 946 Hz, the designed piezoelectric vibrator will produce the maximum output displacement. The bilateral deformation is symmetrical, and the phase difference is zero. Frequency, voltage, and backpressure characteristics of the piezoelectric pump are investigated. The experimental results show that at a certain operating frequency, the flow rate and the backpressure of the piezoelectric pump both increase with the increase in voltage. When the applied voltage is 150 V_pp_, the flow rate reaches a peak of 367.48 mL/min at 720 Hz for one diaphragm pump, and reaches a peak of 700.15 mL/min at 716 Hz for two diaphragm pumps.

## 1. Introduction

Since Thomas et al. first proposed an implantable active-valve piezoelectric pump [1], the development of piezoelectric pumps has been extremely rapid. These pumps work by using the inverse piezoelectric effect of piezoelectric materials to convert electrical energy into mechanical energy. Piezoelectric pumps have many advantages such as compact structure, easy miniaturization, low power consumption, high efficiency, high power density, high control accuracy, fast response, and low noise [2,3,4,5].

In recent years, extensive research has been carried out on the structural design of piezoelectric pumps, mainly focusing on valves [6,7,8], chambers [9,10,11] and vibrators [12,13,14]. Piezoelectric pumps have been widely used in many fields, and research results of significant importance have been achieved. However, the performance requirements for piezoelectric pumps are different in different fields. For example, for applications in the fields of lab-on-a-chip, MEMS, biochemistry, and biomedicine, piezoelectric pumps should have good light transmission performance, biocompatibility or chemical inertness, continuity and stability of fluid control, and high flow and pressure accuracy characteristics. The flow rates are generally in the range of 10 uL/min to 10 mL/min. For fuel supply in aerospace and new energy vehicles fields, and heat dissipation of highly integrated and large-load micro-electronic systems, etc., piezoelectric pumps should have the ability to continuously transport liquid with high flow rates, which are generally in the range of 10 mL/min to 1000 mL/min. And at present, for the study of high flow rate pumps, the flow rates are usually between tens to hundreds mL/min [15,16,17,18,19,20,21,22]. The output characteristics of piezoelectric pumps play a key role in the development of the above fields. The motivation for studying piezoelectric pump in this study is to obtain a pump with a high flow rate to meet the specific requirements of a micro-electronic cooling system, and fuel supply system of aerospace and new energy vehicles.

The maximum deformation of piezoelectric sheet is about one-thousandth to two-thousandth of its size, so the deformation of a single-layer thin piezoelectric sheet is relatively small. Stacking multiple piezoelectric sheets to form a piezoelectric stack is a common method to achieve low-voltage drive and obtain large displacement output at the same time. Valdovinos et al. presented a piezohydraulic pump with a low-voltage piezoelectric stack [15]. The piezohydraulic pump was capable of producing a 125 kPa stall pressure, 186 mL/min no-load flow rate, and 0.14 W of power. Woo et al. designed a reed valve pump using a piezoelectric device as a power source. The achieved pressure and flow rate were 7.96 kPa and 14.95 mL/min, respectively; and the optimum duty was 50% at the high operating frequency [16].

In order to further amplify the displacement of piezoelectric stacks, various types of displacement amplification mechanisms can be used, such as triangular amplification, lever amplification and buckling amplification [17,18]. Ham et al. presented the design, fabrication, and tests of a piezoelectric pump using the hinge-lever amplification mechanism [19]. The pump achieved a no-load flow rate and a maximum output pressure of 600 mL/min and 6.8 kPa, respectively, at the applied voltage of 100 V and driving frequency of 250 Hz. Mohith et al. presented a novel valveless micropump for biomedical applications operated using the amplified piezo vibrator [20]. The proposed micropump could obtain flow rates of 7.192 mL/min, 6.108 mL/min and 5.013 mL/min based on water, blood plasma and whole blood mimicking fluid, respectively.

The combination of resonant drive and piezoelectric stack technology can allow to obtain a high flow rate and a high flow rate density. Ye et al. presented a technique of check valve improvement for high frequency and high flow rate piezoelectric pumps [21]. The proposed technique could obtain a flow rate of 187.2 mL/min at 750 Hz. Wang et al. proposed a square piezoelectric vibrator with a flexible support [22]. The pump chamber diaphragm was separated from the driving unit, and the resonance principle was used to amplify the amplitude of the pump diaphragm. When the pump chamber height was 0.25 mm, the output flow rate of pumping water reached a maximum value of 213.5 mL/min.

In this study, a high flow rate piezoelectric pump is designed, fabricated, and studied to meet the specific requirements of a micro-electronic cooling system, and fuel supply system of aerospace and new energy vehicles. The designed piezoelectric vibrator consists of piezoelectric stacks, a rhombic micro displacement amplifier, and counterweight blocks, which are used to reduce the natural frequency to match with the suitable working frequency of the diaphragm pump. The designed diaphragm pump consists of three parts, a pump chamber, compressible space, and a check valve with 22 bridge check valve units. The high flow rate is obtained with the first vibration displacement superposition of the piezoelectric stack, the second vibration displacement amplification of the displacement amplifier, the third further vibration displacement amplification of the piezoelectric vibrator due to the resonance principle, and the final vibration drive of the pump diaphragm. In order to verify the feasibility of the proposed high flow rate method, the modal analysis of the piezoelectric vibrator is carried out with ANSYS software (Workbench 19.0), and frequency, voltage, and backpressure characteristics of the piezoelectric pump are experimentally investigated and analyzed. 

## 2. Design of the Piezoelectric Pump

### 2.1. Fabrication of the Piezoelectric Stack

At present, the production process of a piezoelectric stack is diverse and is becoming increasingly mature. However, commercial piezoelectric stack products are expensive and have varying types of structures, which do not meet the laboratory’s demand for various developing cross-sections, shapes and sizes. The piezoelectric stack used in this study consists of 26 piezoelectric sheets with dimensions of 10 mm × 10 mm × 0.7 mm (Hunan Jiaye Da Electronic Co., Ltd., Changde, China). The fabrication process is mainly divided into eight steps: (1) select and pretreat piezoelectric sheets; (2) paste the piezoelectric sheets together; (3) sand the piezoelectric stack; (4) coat insulating cement; (5) scratch slots on the insulating cement; (6) connect the positive and negative poles separately; (7) polarize the piezoelectric stack; (8) overall surface insulation treatment.

**Figure 1:**
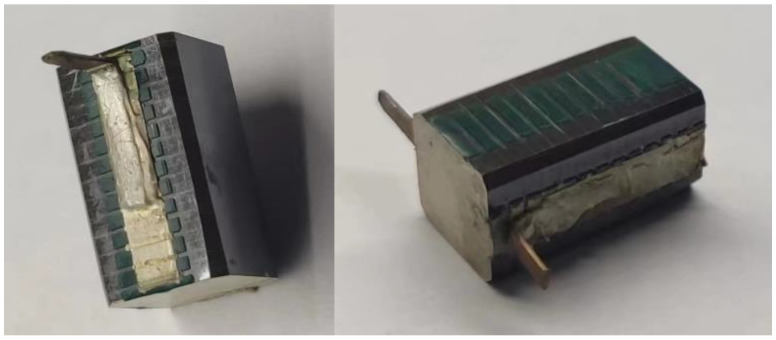
Photograph of the home-made piezoelectric stack.

After completing the above-mentioned 8 steps, a piezoelectric stack with a total length of 18.2 mm is obtained, as shown in Figure 1. The dynamic response and displacement output curves measurement results show that the first resonant frequency of the home-made piezoelectric stack is about 73.05 kHz, and the maximum vibration displacement of the piezoelectric stack is 17.3 μm at the maximum driving voltage of 1400 V_pp_. Here, Vpp refers to the peak-to-peak value of the driving voltage.

### 2.2. Design of the Micro Displacement Amplifier

**Figure 2:**
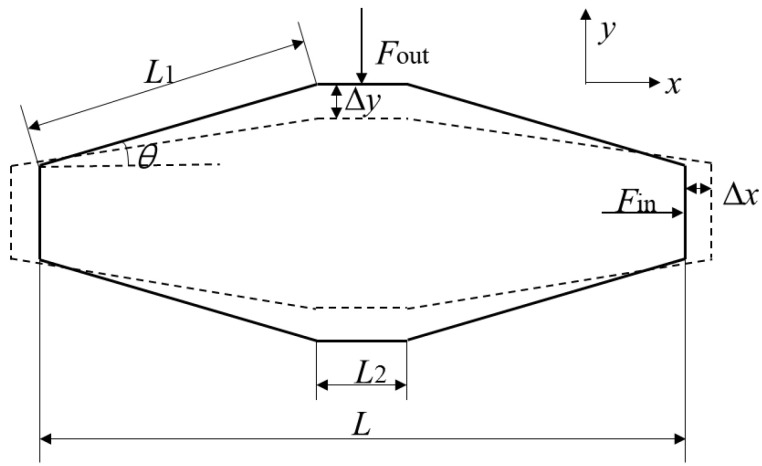
Geometric relationship between the amplifier before and after deformation.

The amplifier designed in this study is a rhombic micro displacement amplifier. Figure 2 shows the geometric relationship between the amplifier before (solid line) and after deformation (dashed line). Two piezoelectric stacks are assembled in the amplifier along the *x* direction. When the piezoelectric stacks are excited by voltage *U*, the output displacement at both ends is ∆*x*, which is transformed into displacement in the *y* direction by the amplifier with the magnitude ∆*y*. The geometric relationship between the input displacement ∆*x* and the output displacement ∆*y* can be expressed as

(1) $${\left(L_{1}\cos\theta +\Delta x\right)}^{2}+{\left(L_{1}\sin\theta -\Delta y\right)}^{2}=L_{1}^{2}$$

where *L*_1_ is the arm length of the amplifier, *θ* is the angle between the arm of the amplifier and the *x* direction, and *L*_2_ is the width of the output end. Therefore, the displacement amplification ratio *M* of the amplifier can be expressed as

(2) $$M=\frac{\Delta y}{\Delta x}=\frac{L_{1}\sin\theta -L1\sqrt{1-{\left(\cos\theta +\frac{\Delta x}{L_{1}}\right)}^{2}}}{\Delta x}$$

When the input displacement ∆*x* is much less than *L*_1_, *M* ≈ cot *θ*. The relationship between the output force *F*_out_ and the input force *F*_in_ can be expressed as

(3) $$F_{\mathrm{out}}/F_{\mathrm{in}}=\Delta x/\Delta y=1/M\approx \tan\theta$$

When the piezoelectric stack without constrains is excited by voltage *U*, the generated force is *KU*, where *K* is the piezoelectric constant related to the strain [23]. Assume that the stiffness of the piezoelectric stack is *k*_0_. Then, the generated displacement can be calculated as

(4) $$k_{0}=\frac{c^{E}A}{L}$$

(5) $$D=KU/(2k_{0})$$

where *c^E^* is the Young’s modulus of the piezoelectric ceramic material; *A* and *L* are the cross-sectional area and length of the piezoelectric stack, respectively.

In fact, when the piezoelectric stack is assembled in the amplifier, which can be called the piezoelectric vibrator, the stiffness of the input end of the amplifier *k*_1_ should be taken into account, which can be estimated using ANSYS software. The actual displacement of the piezoelectric stack ∆*x* can be calculated as

(6) $$\Delta x=KU/(2k_{0}+k_{1})$$

As can be seen from Equation (6), when the piezoelectric stack is assembled in the amplifier, the displacement of the piezoelectric stack will have a certain loss, and the loss will increase with the increase in stiffness *k*_1_. When designing the amplifier, the stiffness of the input end of the amplifier *k*_1_ should be much less than the stiffness of the piezoelectric stack *k*_0_. 

In addition to having a large output displacement, the amplifier also needs to have a large enough output force. It can be seen from Equations (2) and (3) that the smaller the angle *θ*, the larger the displacement amplification ratio *M*, the larger the output displacement, but the smaller the output force. In addition, since two piezoelectric stacks are assembled in the amplifier, the thickness of the amplifier should be slightly larger than the cross-section size of the piezoelectric stack, and the internal longitudinal length of the amplifier should be slightly larger than the total length of the two piezoelectric stacks. Considering the above factors, the geometric parameters of the amplifier are finally obtained; they are shown in Table 1, where *L* is the total length of the amplifier, *T* is the thickness of the amplifier, *t* is the width of the arm, *R* is the radius of the outer fillet, and *r* is the radius of the inner fillet. In this study, the material used for the amplifier is 65 manganese steel (mass density *ρ* = 7840 kg/m^3^, Young’s modulus *E* = 210 Gpa, Poisson ratio *σ* = 0.28, and permissible bending stress [*σ*] = 710 Mpa).

**Figure 3:**
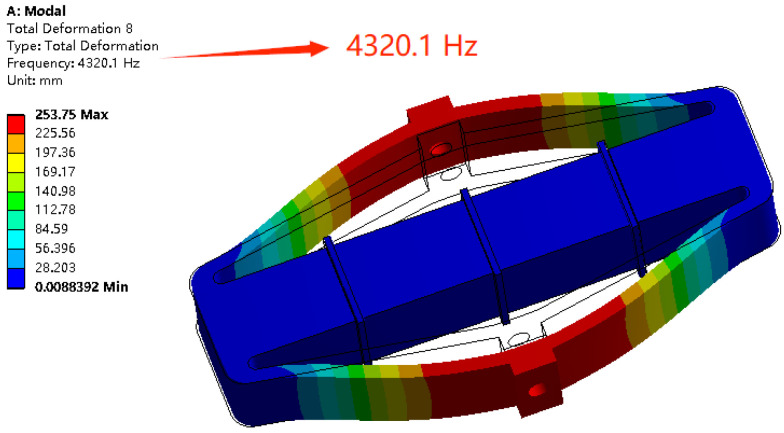
Modal analysis result of the piezoelectric vibrator.

The modal analysis of the piezoelectric vibrator is carried out with ANSYS software. In the simulation, the mesh element size, the number of mesh elements and the number of nodes are 0.5 mm, 304,396 and 97,007, respectively. Figure 3 shows a model diagram of the piezoelectric vibrator whose vibration direction is the same as the output displacement direction at a certain natural frequency (4320 Hz). When the micro displacement amplifier is driven by a piezoelectric stack at the natural frequency, the micro displacement amplifier will produce the maximum output displacement. The bilateral deformation is symmetrical, and the phase difference is zero, which are suitable for symmetrically driving two diaphragm pumps simultaneously. At the same time, the piezoelectric stack will not be subjected to shear force along the *y* direction (Figure 2) at the natural frequency. The piezoelectric stack is therefore not easy to be destroyed due to periodic shear force. Therefore, in this paper, the output of the model at this natural frequency is chosen to obtain the maximum output displacement. 

However, the natural frequency of the piezoelectric vibrator (4320 Hz) is large, and the check valve and pump film used in this study are prone to breakdown and rupture. In this paper, the natural frequency of the piezoelectric vibrator is reduced by increasing the counterweight block, so that it can match with the suitable working frequency of the diaphragm pump (not higher than 1 kHz).

**Figure 4:**
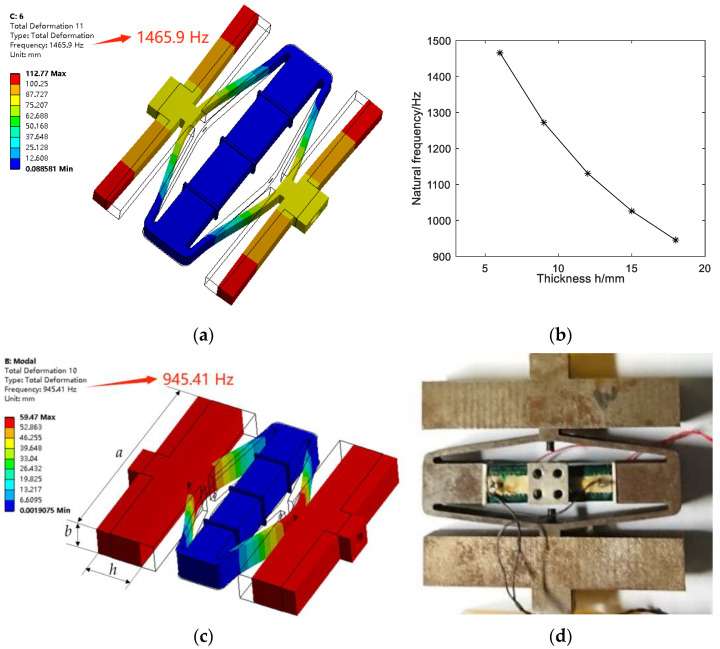
(**a**) Modal analysis result of the piezoelectric vibrator connected to the counterweight block with *h* = 6 mm; (**b**) the relationship between the natural frequency and the thickness of the counterweight block; (**c**) modal analysis result of the piezoelectric vibrator connected to the counterweight block with h = 18 mm; (**d**) assembled piezoelectric vibrator and counterweigh block.

Considering the overall size of the amplifier, the cross-section size of the counterweight block designed in this paper is fixed as *a* × *b* = 78 mm × 12 mm, and only the thickness *h* of the counterweight block is changed. Through simulation analysis, it is found that when the counterweight block is thin, the phenomenon of swaying vibration of the counterweight block will occur (as Figure 4a shows, *h* = 6 mm; the mesh element size, number of mesh elements and number of nodes are 0.5 mm, 385,347 and 143,115, respectively), which will cause unstable operation of the piezoelectric pump, produce loud noise, and cause excess energy loss. When the counterweight block is thick, the counterweight block is not prone to redundant deformation, and the output displacement of the amplifier can be directly transmitted. The natural frequency decreases with the increase in the thickness of the counterweight block, as shown in Figure 4b. Considering that the overall size of the piezoelectric vibrator should be as small as possible, and the natural frequency should be less than 1 kHz, the size of the counterweight block in this paper is set as 78 mm × 12 mm × 18 mm. Figure 4c shows the modal analysis result of the piezoelectric vibrator connected to the counterweight block with *h* = 18 mm. In the simulation, the mesh element size, the number of mesh elements and the number of nodes are 0.5 mm, 443,771 and 177,923, respectively. It can be seen from the figure that the natural frequency (946 Hz) is greatly reduced, which can be used as the reference operating frequency of the piezoelectric pump. The assembled piezoelectric vibrator and counterweigh block is shown in Figure 4d. In order to facilitate the fixation of the piezoelectric vibrator in the experiment, the two piezoelectric stacks are separated by two alumina ceramic sheets with good insulation and high strength. An aluminum alloy connecting the blocks with four screw holes is inserted between the two alumina ceramic sheets. The piezoelectric vibrator can then be fixed on the experimental base with four bolts.

### 2.3. Design of the Diaphragm Pump

**Figure 5:**
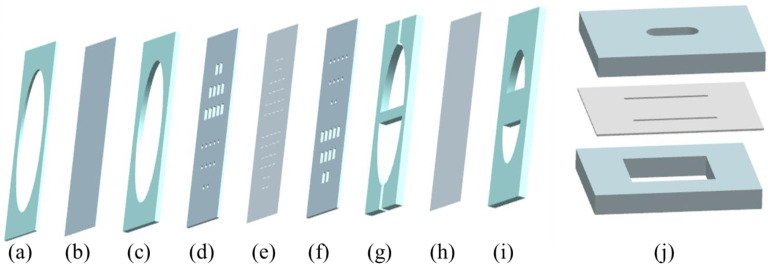
Exploded view of the designed diaphragm pump: (**a**) PMMA fixed plate; (**b**) flexible rubber diaphragm; (**c**) PMMA pump chamber plate; (**d**) PMMA inlet and outlet valve seat plate; (**e**) PDMS check valves; (**f**) PMMA outlet and inlet valve seat plate; (**g**) PMMA flow channels plate; (**h**) PDMS thin film; (**i**) PMMA compressible spaces plate; (**j**) check valve unit.

The exploded view of the designed diaphragm pump is shown in Figure 5. The diaphragm pump consists of three parts: pump chamber, check valve and compressible space. One unit of the check valve is shown in Figure 5j. One PMMA board has a long hole consisting of a rectangle and two semicircles, while the other has a large rectangular hole. When two narrow gaps in the PDMS film along the long edge of the large rectangular hole are cut, the formed bridge valve can completely cover the long hole, so that the liquid can pass through the valve from the long hole to the large rectangular hole, but is blocked in the other direction.

For the components of the designed diaphragm pump, shown in Figure 5, the material used for processing (a), (c), (d), (f), (g) and (i) is PMMA, which has the characteristics of high mechanical strength, high transparency, easy operation, and low price. During the experiment, the pump is not easy to deform, and the fluid flow in the pump can be observed. The material of (e) and (h) is PDMS, which has the characteristics of high resilience and elasticity, high light transmittance, and has a good electrostatic adhesion effect. During the experiment, the valve would have a long service life, and will be easy to cut and assemble. Material (b) is a flexible rubber diaphragm, which is a highly elastic polymer material with reversible deformation. It is elastic at room temperature, and can produce large deformation under the action of a small external force. During the experiment, the pump would have a long service life and will be easy to compress.

The designed diaphragm pump has a square cross-section. First, one piece of the machined PMMA inlet and outlet valve seat plate, one piece cut from the square PDMS thin film, and another piece of the PMMA inlet and outlet valve seat plate are aligned on top of each other. Note that the large rectangular holes in one piece of PMMA inlet and outlet valve seat plate should correspond to the long holes in the other piece, and the air bubbles between them must be removed. Narrow gaps are then carved in the corresponding positions of the PDMS film to form 22 bridge check valves, which can meet the needs of the large flow rate of liquids. Then, according to the corresponding relationship shown in Figure 5, one piece of the PMMA flow channels plate, one piece cut from the square PDMS thin film, and one piece of the PMMA compressible spaces plate are aligned on top of each other successively to create the compressible space. In this study, the main reason for choosing the PDMS thin film to create compressible spaces instead of the PMMA plate to seal directly is that the compressible space can reduce the dynamic load of fluid mass and improve pump performance. On the other side of the check valve, one piece of the PMMA pump chamber plate, one piece cut from the square flexible rubber diaphragm, and one piece of the PMMA fixed plate are aligned on top of each other successively to create a pump chamber. The inlet pipe and outlet pipe are respectively inserted into the inlet and outlet of the PMMA flow channels plate. Finally, the diaphragm pump is sealed using the epoxy adhesive (3M™ Scotch-Weld™, DP460) with good shear and peel strengths along with good impact and durability. After a sealing test, the performance of the designed piezoelectric pump can be tested.

**Figure 6:**
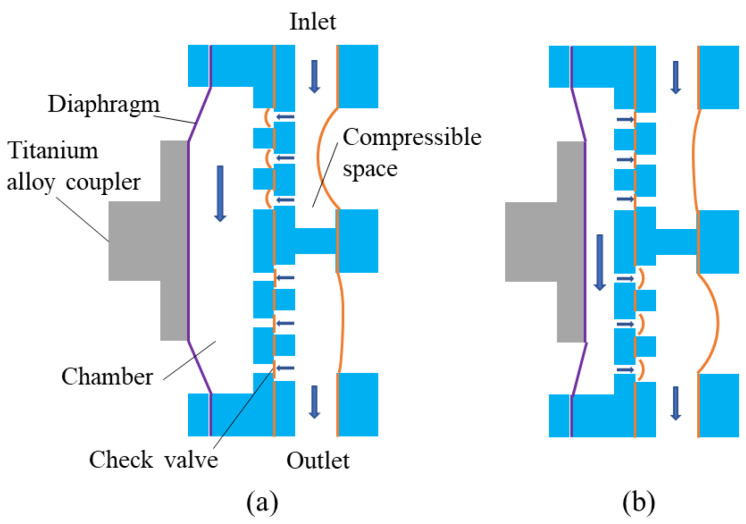
Operation principle of the designed diaphragm pump: (**a**) supply mode; (**b**) pump mode.

The operation principle of the designed diaphragm pump is shown in Figure 6. When a driving voltage is applied, two piezoelectric stacks convert the electrical energy into mechanical vibration energy. The two piezoelectric stacks are assembled in the displacement amplifier, so the vibrations on the piezoelectric stacks along the *x* direction (Figure 2) are transmitted to the input ends of the amplifier, and then transmitted to the output ends after amplification by the amplifier. The output vibration is then transmitted to the flexible rubber diaphragm of the diaphragm pump through the counterweight block and a titanium alloy coupler. When the distance between the two output ends of the piezoelectric vibrator is shortened due to vibration (Figure 6a), the titanium alloy coupler will pull the pump diaphragm away from the pump chamber, resulting in an increase in the volume of the pump chamber. Consequently, the pressure in the chamber decreases as the volume of the chamber increases, the inlet valve units are opened, the outlet valve units are closed, and the liquid in the inlet is drawn into the pump chamber through the opened inlet valves. When the distance between the two output ends of the piezoelectric vibrator is elongated due to vibration (Figure 6b), the titanium alloy coupler will push the diaphragm towards the chamber, resulting in a decrease in the volume of the pump chamber. Consequently, the pressure in the chamber increases as the volume of the chamber decreases, the inlet valve units are closed, the outlet valve units are opened, and the liquid in the chamber is squeezed out into the outlet through the opened outlet valves. The diaphragm pump realized the conversion of mechanical vibration energy of the piezoelectric vibrator into fluid flow energy. When driven by a periodic alternating current voltage, the two modes in Figure 6a,b will be alternated to achieve continuous liquid delivery from the inlet to the outlet. 

## 3. Experimental Results and Discussion

### 3.1. Experimental Setup

**Figure 7:**
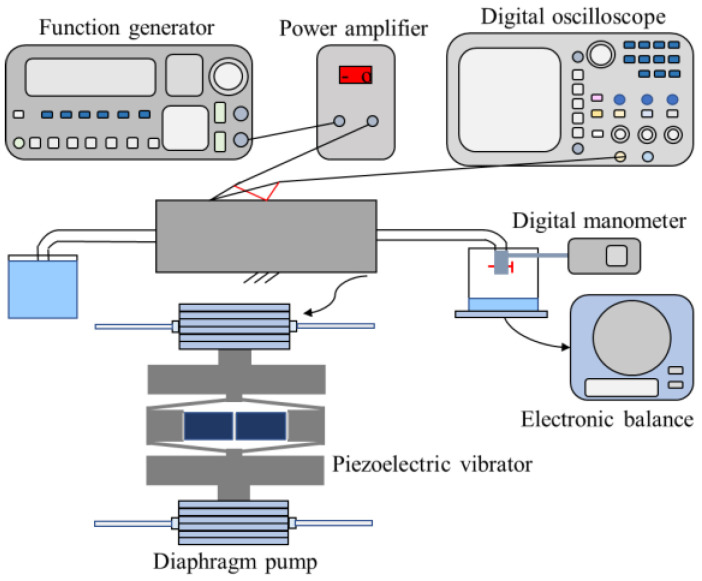
Schematic illustration of the experimental setup.

In this paper, the performance of a designed piezoelectric pump is tested, and the experimental setup is shown in Figure 7. A function generator (DG1022U, RIGOL, Beijing, China) and a power amplifier (LYF-800AS, Lykj, Nantong, China) are used to generate electrical signals of sufficient strength and appropriate frequency for the piezoelectric vibrator. A digital oscilloscope (TBS 1000X Series, Tektronix, Beaverton, OR, USA) is used to monitor the frequency and amplitude of the input waveform. Electronic balance is used to weigh the amount of liquid pumped in a certain period of time so as to calculate the flow rate of the liquid. A digital manometer (HT-1895, XINTEST, Shenzhen, China) is used to measure the backpressure of the diaphragm pump. Tap water is utilized in the experiment. 

In the experiment, firstly, the piezoelectric vibrator is fixed on the experimental base with bolts. Then, the position of the diaphragm pump is adjusted and fixed with a clamp to match the piezoelectric vibrator. The case of driving two diaphragm pumps is shown in Figure 7. Then, the circuit is connected. After the function generator, power amplifier, digital oscilloscope, and the positive and negative electrodes of the piezoelectric stacks are correctly connected, the water tank, electronic balance, and digital manometer are placed in appropriate positions. The performance of the piezoelectric pump can then be tested.

### 3.2. Results and Discussion

In order to avoid the difference in experimental results caused by factors such as asymmetry during testing, the performance of one diaphragm pump is first tested in this paper. Frequency characteristics of the pump is investigated first. During the experiment, in order to prevent the sudden and violent vibration of the piezoelectric vibrator from damaging the diaphragm pump, low-frequency and low-voltage electrical signal should be first input into the piezoelectric pump. Then, the frequency should be gradually adjusted to find the frequencies when the diaphragm pump begins to pump water and the flow rate is significantly reduced. Frequency characteristics test is then performed between these two frequencies.

**Figure 8:**
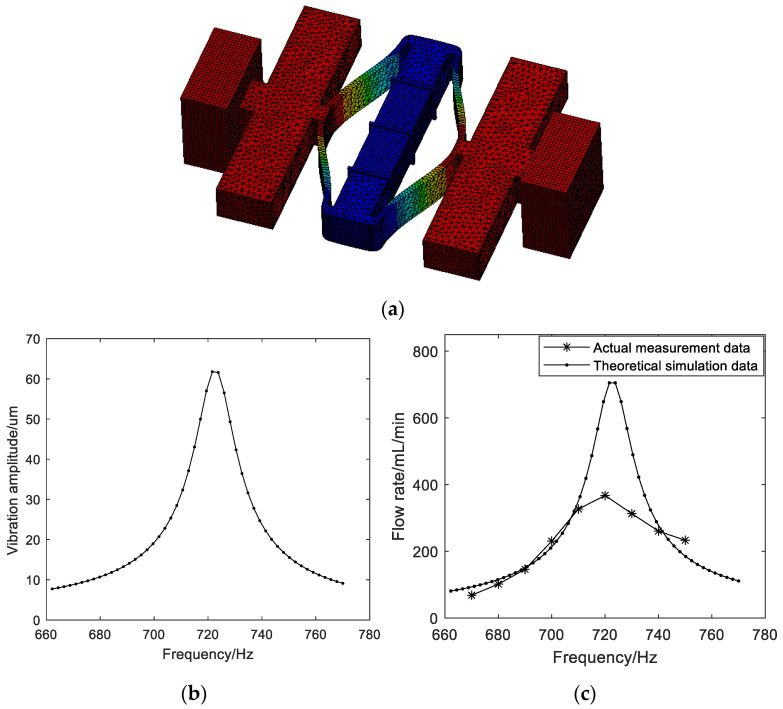
(**a**) Schematic of the model for the piezoelectric pump; (**b**) the relationship between the theoretical vibration amplitude of the pump diaphragm and the input frequency; (**c**) the relationship between the output flow rate and the input frequency.

When the voltage of the electrical signal is 150 V_pp_, the measured relationship between the output flow rate and the input frequency is shown in Figure 8c. The diaphragm pump begins to pump water appreciably at 670 Hz. At low frequencies, the flow rate increases with the increase in frequency. Then, a decreasing slope results in the maximum flow rate of 367.48 mL/min at 720 Hz. At high frequencies, the flow rate decreases with the increase in frequency. 

For the designed piezoelectric pump, the diameters of the pump chamber and the titanium alloy coupler are constant. According to reference [24], there is *Q*_t_∝*A*_m_ × *f*, where *Q*_t_ is the theoretical flow rate, *f* is the input frequency, and *A*_m_ is the vibration amplitude of the pump diaphragm which is equal to the output amplitude of the piezoelectric vibrator *A*_out_. To evaluate the relationship between the theoretical flow rate and the input frequency, firstly, the harmonic analysis of the piezoelectric pump is executed using ANSYS software. The schematic of the model for the piezoelectric pump is shown in Figure 8a. Here, an assumption is made that the diaphragm pumps are equivalent to two mass blocks. The frequency response curve at the monitoring point obtained via harmonic analysis, that is, the relationship between the theoretical displacement vibration amplitude of the pump diaphragm and the input frequency, is shown in Figure 8b. For the case of resonant drive, the amplitude has a peak value at the resonant frequency due to the resonance principle. Finally, the variation in the theoretical flow rate with the input frequency is calculated, as shown in Figure 8c. Because experimental conditions are idealized or simplified in the theoretical derivation and calculation, there are differences between the theoretical and actual data. However, the theoretical and actual trends of flow rate variation with frequency are consistent.

**Figure 9:**
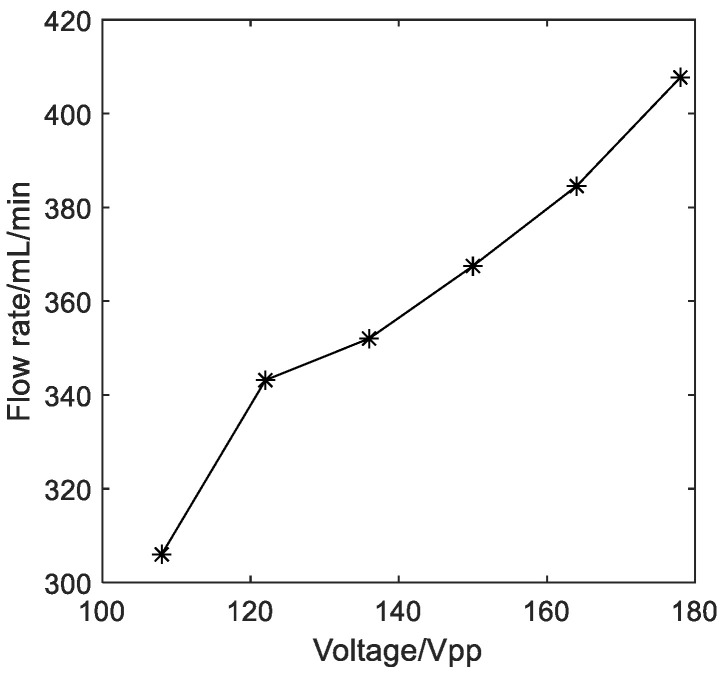
The relationship between the output flow rate and the input voltage.

Then, the relationship between the output flow rate of the diaphragm pump and the voltage of the input electrical signal is tested at the frequency of 720 Hz. During the experiment, the voltage first gradually increases to the maximum value, and then gradually decreases, and the average of the two measured values at the same voltage is the resultant flow rate. The relationship between the output flow rate and the input voltage is shown in Figure 9. It can be seen that the greater the voltage value of the input signal, the greater the output flow rate of the diaphragm pump. The flow rate of the pump reaches 407.7 mL/min at the applied voltage of 178 V_pp_. Theoretically, *k*_0_, *k*_1_ and *K* are constant for the piezoelectric pump that has been designed. According to Equation (6), the input amplitude of the piezoelectric vibrator *A*_in_ is proportional to the input voltage *U*, that is, *A*_in_∝*U*. According to Equations (2) and (3), the output amplitude of the piezoelectric vibrator *A*_out_ is also proportional to the input voltage *U*, that is, *A*_out_∝*U*, and there is *A*_m_∝*U*. When the input frequency is constant, there is *Q*_t_∝*A*_m_ × *f*∝*A*_m_∝*U*; that is, the theoretical flow rate is proportional to the input voltage. The approximately linear relationship between the output flow rate and the input voltage in the measurement range shown in Figure 9 is consistent with this theoretical derivation.

**Figure 10:**
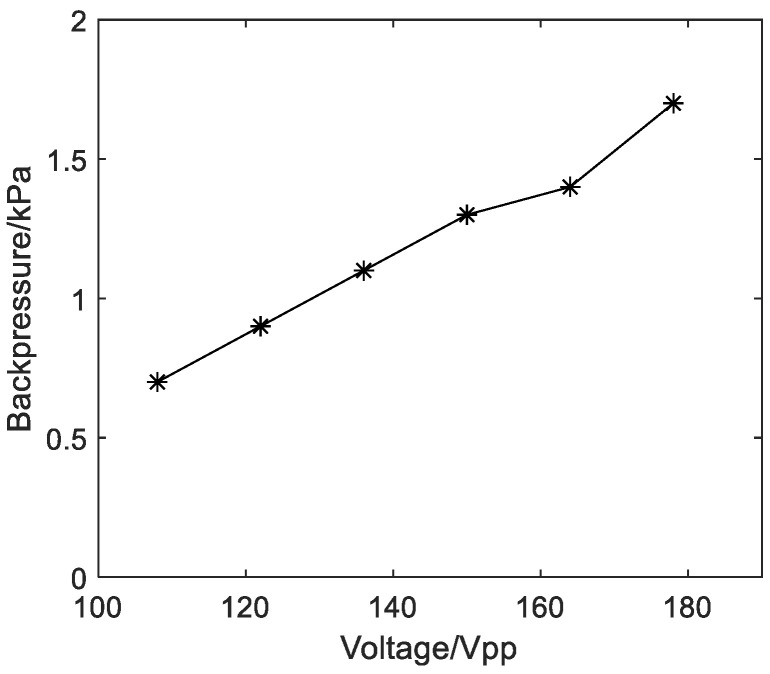
The relationship between the backpressure and the input voltage.

When measuring the backpressure, a connecting tube is used to connect the outlet pipe of the pump and the digital manometer (Figure 7). The backpressure of the pump can then be obtained, which is the value displayed on the digital manometer. At the frequency of 720 Hz, the relationship between the backpressure of the diaphragm pump and the voltage of the input electrical signal is shown in Figure 10. It should be noted that the measurement process should be rapid; otherwise, the film of the pump chamber may break due to excessive pressure. It can be seen that the greater the voltage value of the input electrical signal, the greater the backpressure of the diaphragm pump. The backpressure of the pump is 1.7 kPa at the applied voltage of 178 V_pp_. It can also be seen from the above results that at a certain operating frequency, when the pressure in the pump chamber increases, the output flow rate also increases. 

Finally, the maximum flow rate of two diaphragm pumps is tested. Experimental results show that when the voltage of the electrical signal is 150 V_pp_, at about 716 Hz, the flow rate reaches a peak of 700.15 mL/min. The proposed piezoelectric pump in this study does possess a larger flow rate compared with several recently reported works in terms of maximum flow rate [16,19,20,21,22].

## 4. Conclusions

In order to achieve high output performance, a resonantly driven piezoelectric pump with one or two diaphragm pumps and a piezoelectric vibrator is presented in this paper. The designed diaphragm pump consists of a pump chamber, a check valve and a compressible space. The compressible space of the diaphragm pump contains a piece of PDMS thin film, which can reduce the dynamic load of fluid mass and improve pump performance. The check valve contains 22 bridge check valve units, which can meet the needs of the large flow rate of liquid. The designed piezoelectric vibrator is composed of piezoelectric stacks and a micro displacement amplifier. In order to reduce the natural frequency to match with the suitable working frequency of the diaphragm pump, counterweight blocks are added. The natural frequency of the piezoelectric vibrator with symmetrical vibration modes and the maximum output displacement is selected as a reference, so that the two diaphragm pumps can be symmetrically driven simultaneously. Frequency, voltage, and backpressure characteristics of the piezoelectric pump are investigated. The measurement results show that the proposed piezoelectric pump in this study does possess a larger flow rate compared with several recently reported works in terms of maximum flow rate. In addition, the obtained flow rate meets the high flow rate requirement of micro-electronic cooling systems, and fuel supply systems of aerospace and new energy vehicles. However, the overall size of the piezoelectric pump designed in this paper is larger. Therefore, our further work will aim to optimize the designed piezoelectric pump to obtain a more compact pump.

## Figures and Tables

**Table 1:** Geometric parameters of the amplifier.

*L*	*θ*	*L*_1_	*L*_2_	*t*	*T*	*R*	*r*
78 mm	10⁰	34 mm	6 mm	11 sin(10⁰) mm	12 mm	3 mm	1 mm

## Data Availability

The data presented in this study are available upon reasonable request from the authors.
